# Establishment of a 3D spheroid culture system to evaluate the responsiveness of uterine leiomyoma cells to female hormones

**DOI:** 10.1002/rmb2.12627

**Published:** 2025-01-22

**Authors:** Takahiro Sakai, Shun Sato, Tetsuro Tamehisa, Hitomi Takasaki, Takuya Kajimura, Isao Tamura, Norihiro Sugino

**Affiliations:** ^1^ Department of Obstetrics and Gynecology Yamaguchi University Graduate School of Medicine Ube Japan

**Keywords:** female hormones, mediator complex subunit 12 (MED12) mutation, selective progesterone receptor modulator, spheroid, uterine leiomyomas

## Abstract

**Purpose:**

Uterine leiomyomas (ULMs) are classified into those with and without MED12 mutations (MED12m(+) and MED12m(−), respectively). This study was undertaken to establish a culture system to evaluate the effect of female hormones on the growth of ULM cells in each ULM subtype.

**Methods:**

ULM cells isolated from MED12m(+) or MED12m(−) tissues were cultured in a monolayer for 7 days with four hormone treatments: estrogen (E) and progesterone (P) (E + P), E only (E), P only (P), and medium only (CTRL). They were also cultured in a 3D spheroid culture system with the above four treatments and a fifth treatment: E + P + selective progesterone receptor modulator (E + P + SPRM). The hormonal effects were evaluated based on cell number, spheroid size, and histology.

**Results:**

In the monolayer cultures, female hormones did not cause the proliferation of ULM cells of either subtype. In the spheroid cultures, spheroid sizes for both subtypes were significantly larger with the E + P and P treatments than with the CTRL and E treatments and were comparable in the E and E + P + SPRM treatments. Histological staining showed that collagen fibers were present only in the spheroids of the P‐treated groups of MED12m(+).

**Conclusion:**

We established a 3D spheroid culture system to evaluate the effects of female hormones on ULM cells.

## 1 INTRODUCTION

Uterine leiomyomas (ULMs) are the most frequent gynecological tumors originating from smooth muscle cells (SMCs) in the myometrium.^1^ Although ULMs are benign diseases, they cause severe menstrual pain, anemia, infertility, and miscarriage, thus impairing the quality of life of women.^1^, ^2^ Surgical treatment such as hysterectomy or myomectomy is essential to cure ULMs. However, with the trend toward late marriages and the aging of childbearing due to recent lifestyle changes, there is a growing demand for drug therapy that can preserve the uterus. The development of effective drugs requires elucidating the pathogenesis of ULMs and validating the drug efficacy, which requires a model system that reflects the characteristics of ULMs in vivo.

Missense mutations in the mediator complex subunit 12 (*MED12*) gene have been found in ULMs as a driver mutation involved in their pathogenesis, and the mutations were detected in about 70% of ULM specimens regardless of ethnicity.^3^, ^4^, ^5^, ^6^, ^7^, ^8^, ^9^, ^10^ Therefore, ULMs are classified into at least two subtypes with different pathogeneses depending on the presence or absence of *MED12* mutations. Recently, the histological composition, including collagen fiber content and cellular components, was reported to differ between the ULM subtypes.^11^, ^12^ We also reported that ULMs with *MED12* mutation (MED12m(+)‐ULM) contain abundant collagen fibers and are composed of approximately 60% SMCs and 40% fibroblasts (FBs), whereas ULMs without *MED12* mutation (MED12m(−)‐ULM) contain few collagen fibers and are composed of approximately 80% SMCs and 20% FBs.^12^ Studies using the xenograft model system showed that SMCs and FBs differ in their responsiveness to female hormones and that FBs can proliferate with estrogen (E) alone while SMC proliferation requires both E and progesterone (P).^11^, ^13^ In clinical research, the tumor reduction effect of gonadotropin‐releasing hormone analog (GnRHa), which suppresses E and P secretion, was higher in MED12m(−)‐ULM tissues than that in MED12m(+)‐ULM tissues, indicating that the two ULM subtypes are differently affected by withdrawal of E and P.^14^ These findings suggest that the two ULM subtypes respond differently to female hormones.

So far, most studies on the effect of female hormones on the growth of ULMs have been conducted in 2D monolayer cultures. Some studies reported that female hormones or their agonists increased the proliferation of ULM cells in 2D cultures,^15^, ^16^, ^17^, ^18^ while others reported that female hormones did not cause the proliferation of ULM cells.^19^, ^20^, ^21^ Thus, the responsiveness of ULM cells to female hormones in a 2D monolayer culture remains unclear. Furthermore, in many of these studies, ULM subtypes were not distinguished because the concept of the subtypes was not yet established. It was also reported that in a 2D monolayer culture of ULM cells, FBs occupy the majority of ULM cells during long‐term cultures.^22^, ^23^ Therefore, it remains unclear whether the effect of female hormones was from SMCs or FBs. In addition, maintaining the original characteristics of the ULM cells in 2D monolayer cultures is difficult because, in in vitro culture, there is a rapid loss in the expression of estrogen receptor 1 (*ESR1*) and progesterone receptor (*PGR*), which alters gene expression profiles in ULM cells.^24^, ^25^ Although there are several studies reporting that a 3D spheroid culture system of ULM cells more closely reflects the in vivo characteristics compared with a 2D monolayer culture,^26^, ^27^, ^28^ they do not investigate the effect of female hormones and the difference between the ULM subtypes.

In this study, in addition to examining the effect of female hormones on ULMs, we investigated the effect of ulipristal acetate (UPA), a selective progesterone receptor modulator (SPRM) that inhibits PGR function,^29^ to further examine the PGR‐mediated effect of P on ULMs. In 2D monolayer cultures, UPA has been shown to induce apoptosis,^30^ decrease collagen fiber production,^31^ and suppress the expression of vascular endothelial growth factor and its receptor.^32^ However, these studies did not differentiate the two ULM subtypes.

The present study was undertaken to investigate the effect of female hormones on the growth of ULM cells derived from MED12m(−)‐ and MED12m(+)‐ULM tissues in 2D monolayer and 3D spheroid cultures after we identified components of the cells and measured their expressions of female hormone receptors. We eventually established a 3D spheroid culture system that can evaluate the responsiveness of ULM cells to female hormones.

## 2 METHODS

### 2.1 Patient tissue samples

For this study, ULM specimens were obtained from 18 Japanese women who underwent hysterectomy (Table 1). Specimens of MED12m(−)‐ULM were obtained from nine of the women (aged 34–51 years, mean ± SD; 45.9 ± 5.3 years). Specimens of MED12m(+)‐ULM were obtained from 11 of the women (aged 32–54 years, mean ± SD; 46.0 ± 7.1 years). Two of the women provided both MED12m(−)‐ULM and MED12m(+)‐ULM. None of the women had received previous treatment with female hormones or GnRHa. This study was reviewed and approved by our hospital's Ethical Review Committee for Medical Research for collecting tissue specimens. Written informed consent was obtained from all patients before collecting the specimens.

**TABLE 1:** Basic information in the ULM specimens used for the monolayer or the spheroid culture system in this study.

ULM ID	MED12 mutation	Age	Major axis × minor axis (mm × mm)	Type	Surgical method	Collagen fiber rate (%)	SMC rate (%)
MED12m(−)‐ULM							
Monolayer‐culture							
#44	–	46	45 × 39	Submucosal	TLH	24.5	81.8
#47	–	48	67 × 53	Intramural	TLH	16.6	89.2
#62–1	–	50	77 × 64	Intramural	TLH	19.1	76.8
#63	–	48	192 × 78	Intramural	AT	16.1	85.5
Mean ± SD		48.0 ± 1.6				19.1 ± 3.8	83.3 ± 5.3
Spheroid‐culture							
#65	–	46	71 × 70	Submucosal	RAH	22.1	75.3
#68–1	–	51	67 × 58	Intramural	RAH	17.9	82.7
#69	–	49	37 × 27	Intramural	TLH	20.6	89.0
#73	–	34	48 × 44	Intramural	LM	11.1	87.1
#86	–	41	34 × 30	Submucosal	LM	4.7	87.7
Mean ± SD		44.2 ± 6.8				15.3 ± 7.3	84.4 ± 5.6
Total							
Mean ± SD		45.9 ± 5.3				17.0 ± 6.0	83.9 ± 5.1
MED12m(+)‐ULM							
Monolayer‐culture							
#52	c.131G > A	37	36 × 36	Intramural	LM	27.5	69.6
#55	c.130G > A	44	42 × 29	Intramural	TLH	38.4	65.7
#60	c.130G > A	49	42 × 40	Submucosal	RAH	52.6	69.8
#62–2	c.131G > T	50	39 × 38	Intramural	TLH	43.1	62.7
Mean ± SD		45.0 ± 7.2				40.4 ± 12.7	67.0 ± 3.4
Spheroid‐culture							
#68–2	IVS1‐8 T > A	51	35 × 32	Intramural	RAH	43.1	67.4
#70	c.[130G>A; 131G>A]	49	42 × 36	Intramural	AT	56.9	62.7
#72	c.126_149del24	32	90 × 70	Subserous	LM	41.0	62.2
#74–1	c.131G>A	39	100 × 71	Intramural	LM	29.7	‐
#74–2	c.131G>A	39	28 × 23	Intramural	LM	43.1	54.0
#76	c.131G>T	52	23 × 15	Intramural	AT	25.1	59.0
#82–1	+*	54	25 × 25	Intramural	RAH	44.9	62.7
#82–2	c.124_144del21	54	35 × 27	Intramural	RAH	46.0	68.6
#84	c.130G>A	48	31 × 20	Intramural	RAH	47.8	68.6
Mean ± SD		46.4 ± 7.9				42.0 ± 9.5	63.2 ± 5.1
Total							
Mean ± SD		46.0 ± 7.1				41.5 ± 9.4	64.4 ± 4.8

Abbreviations: AT, abdominal simple total hysterectomy;LM, laparoscopic myomectomy; RAH, robot‐assisted simple total hysterectomy; SMC, smooth muscle cell; TLH, laparoscopic total simple hysterectomy.

* A specimen that had a deletion mutation but could not be sequenced. Collagen fiber rate (%) and SMC rate (%) were calculated as reported previously (ref. #12).

### 2.2 Genomic DNA isolation and *MED12* genomic sequencing

Genomic DNA from the ULM specimens was isolated as reported previously.^33^, ^34^, ^35^, ^36^, ^37^ In brief, the genomic DNA was isolated by treatment with proteinase K (Qiagen, Hilden, Germany), followed by phenol/chloroform extraction and ethanol precipitation.

The exon 2 region of the *MED12* gene in the ULM specimens was sequenced as reported previously.^3^, ^8^, ^9^, ^10^ In brief, genomic PCR was performed using 10 ng genomic DNA, 1.25 units of PrimeSTAR GXL DNA polymerase (Takara, Kyoto, Japan), and a primer set as follows: 5'‐GCCCTTTCACCTTGTTCCTT‐3′ and 5'‐TGTCCCTATAAGTCTTCCCAACC‐3′ under the thermocycling conditions (35 cycles of 98°C for 10 s, 60°C for 15 s, and 68°C for 20 s). The amplified PCR products were purified using a PCR purification kit (Qiagen) and sequenced using the BigDye Terminator v3·1 Cycle Sequencing Kit (Applied Biosystems, Carlsbad, CA) by the ABI PRISM 3130xl Genetic Analyzer. The obtained sequence chromatograms were analyzed manually.

### 2.3 Preparation of ULM cells and 2D monolayer and 3D spheroid cultures

The preparation of ULM cells from ULM specimens was performed according to the protocol of Kurita et al.^38^ Briefly, ULM specimens were cut into 1–2 mm square pieces with a razor and treated with 5‐fold amounts of 1.5 mg/mL collagenase Type I (Wako, Osaka, Japan), 10 μg/mL Dnase I (Roche, Basel, Switzerland), and 1% antibiotic‐antimycotic (Wako) in HBSS, and incubated at 37°C for 6 h with shaking at 250 rpm. The ULM cells were collected through a 100 μm cell strainer (Falcon, Corning, NY) to remove the debris and counted on a TC20 automated cell counter (BioRad, Berkeley, CA). The ULM cells were then seeded at 2 × 10^6^ cells in 10 cm collagen‐coated dishes (Corning, Corning, NY) and maintained in DMEM/F12 (Wako) containing 10% FBS (Gibco, Rockville, MD), 2 mM L‐glutamine (Gibco), and 1% antibiotic‐antimycotic (Wako) at 37°C in a humidified atmosphere of 5% CO_2_ and 95% air.

**FIGURE 1:**
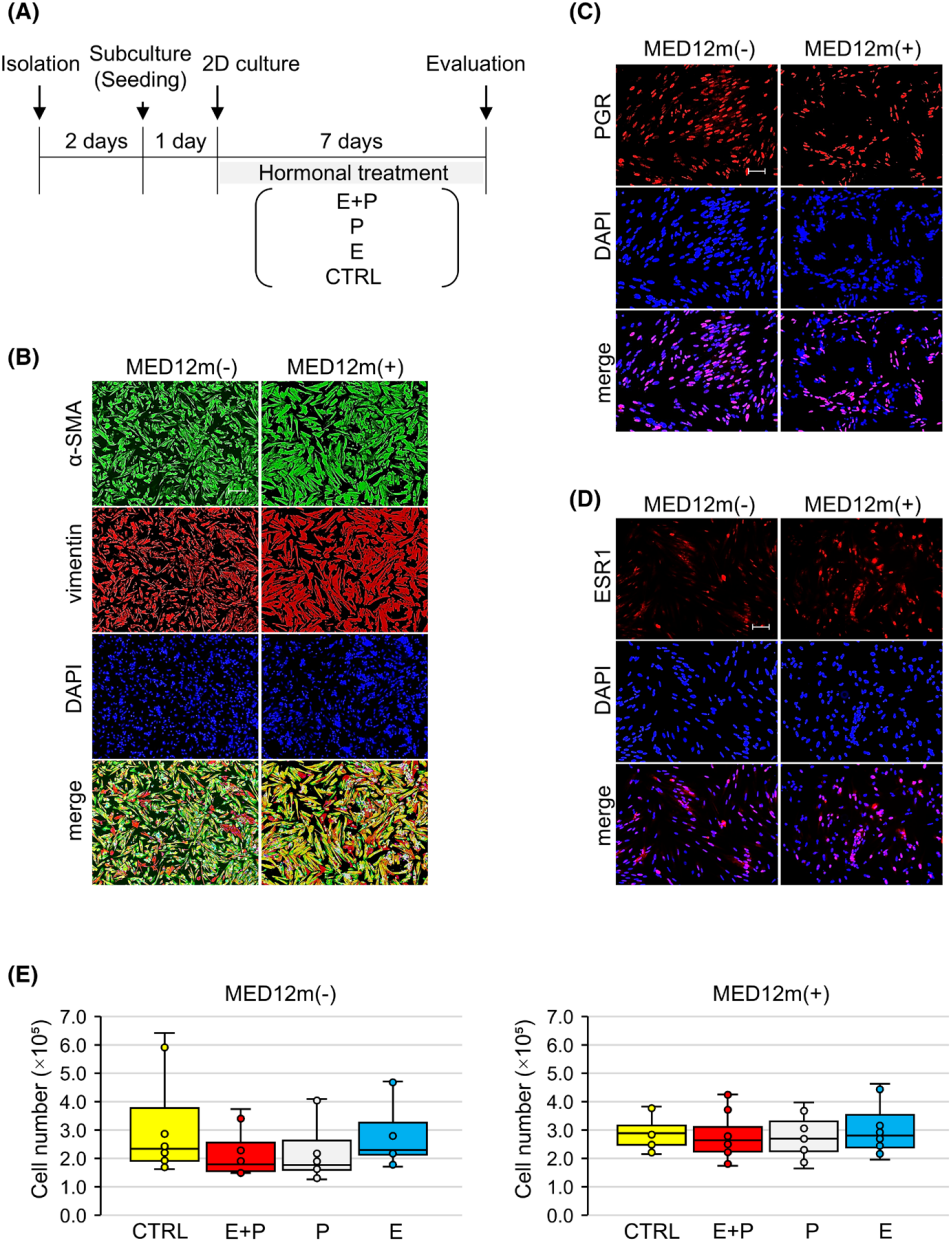
Effects of female hormones on uterine leiomyoma (ULM) cells in 2D monolayer cultures. (A) Schematic diagram of female hormone treatment experiments. ULM cells 2 days after isolation were subcultured and then treated with female hormones in four groups: estrogen (E) + progesterone (P) (E + P), P alone (P), E alone (E), and medium (CTRL), for 7 days. The concentrations of E and P were 10^−8^ M and 10^−6^ M, respectively. The effect of female hormones was evaluated by cell proliferation assay. (B, C) Immunocytochemical staining images of MED12m(−)‐ and MED12m(+)‐ULM cells at the 2D culture (3 days after isolation) for α‐SMA (a SMC marker; green) and vimentin (a mesenchymal cell marker; red) (B), PGR (C), and ESR1 (D), respectively. Nuclear staining was performed with DAPI. Scale bars = 50 μm. (E) Incubations were performed in triplicate in a specimen from each ULM subtype, and the cell number in each well was calculated. Four incubations from 4 specimens were performed, and box plots of cell numbers are shown as mean ± SD of 4 incubation.

For all ULM cells in the 2D monolayer and 3D spheroid cultures, cellular components and expression of female hormone receptors were examined on day 3 of primary culture by immunocytochemistry as described in METHODS 2.5 below. Almost all ULM cells were vimentin‐positive mesenchymal cells (Figure 1B). The percentage of α‐SMA (a SMC marker) positive cells (Figure 1B) averaged 83.7% ± 7.6% and 82.7% ± 6.7% in MED12m(−)‐ and MED12m(+)‐ULM, respectively, indicating that SMCs are the majority of the ULM cells. The percentage of the PGR‐positive cells (Figure 1C) and ESR1‐positive cells (Figure 1D) were as follows: PGR‐positive: 36.5% ± 13.4% in MED12m(−)‐ULM and 47.2% ± 11.2% in MED12m(+)‐ULM; ESR1‐positive: 16.7% ± 4.4% in MED12m(−)‐ULM and 24.7% ± 15.3% in MED12m(+)‐ULM. There were no significant differences in α‐SMA, PGR, or ESR1‐positive cells between both subtypes.

For 2D monolayer cultures (Figure 1A), 2 days after isolation, MED12m(−)‐ and MED12m(+)‐ULM cells were subcultured at concentrations of 2 × 10^5^ cells/well and 5 × 10^5^ cells/well, respectively, in a 12‐well plate coated with 0.5% gelatin. One day after subculture, the ULM cells were treated with female hormones for 7 days as follows: estrogen (β‐estradiol: Sigma, St. Louis, MO) (E) + progesterone (progesterone: Fujifirm, Tokyo, Japan) (P) (E + P), P alone (P), E alone (E), and medium (CTRL).

**FIGURE 2:**
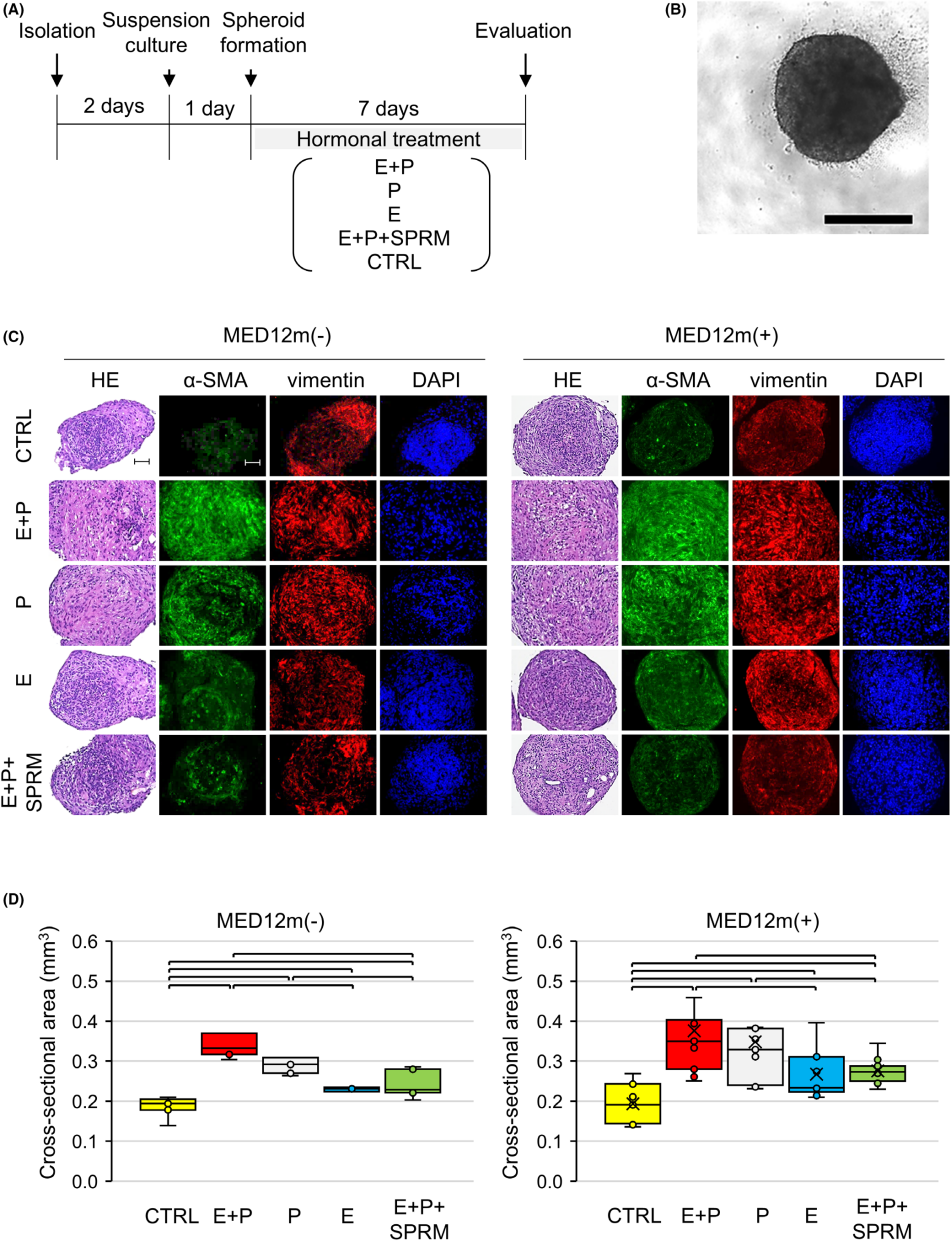
Effects of female hormones on ULM cells in 3D spheroid cultures. (A) Schematic diagram of female hormone treatment experiments. ULM cells 2 days after isolation were suspension‐cultured for 24 h, and the spheroid formation was confirmed. Then, the spheroids were treated with female hormones in five groups: E + P, P, E, CTRL, and E + P + SPRM for 7 days. The concentrations of E, P, and SPRM were 10^−8^ M, 10^−6^ M, and 10^−7^ M, respectively. The effect of female hormones was assessed by spheroid size and histology. (B) A representative of a ULM spheroid formed after 1 day of suspension culture in MED12m(−)‐ULM cells. Scale bar = 500 μm. (C) HE and immunohistochemical staining with α‐SMA (green) and vimentin (red) in MED12m(−)‐ and MED12m(+)‐ULM spheroids after 7 days of hormone treatments. Nuclear staining was performed with DAPI. Scale bars = 50 μm. (D) The largest cross‐sectional area of the spheroid was evaluated as a size of the spheroid., imaged with a Keyence BZ‐X710 microscope (Keyence, Osaka, Japan), and measured with ImageJ. Six spheroids were made from a specimen of ULMs. The mean of 6 spheroids was a value of a specimen. Box plots are shown as the mean of 5 specimens of MED12m(−)‐ULM and 9 specimens of MED12m(+)‐ULM. Brackets in the box plots indicate significant differences between the culture groups (*p*‐value < 0.05, Mann–Whitney test).

For 3D spheroid cultures (Figure 2A), 2 days after isolation, MED12m(−)‐ and MED12m(+)‐ULM cells were suspension‐cultured at 5 × 10^4^ cells/well on an ultra‐low attachment 96‐well dish (Corning). Spheroid formation was confirmed 24 h after suspension culture (Figure 2B). The spheroids were treated with E + P, P, E, CTRL, and E + P + SPRM (UPA: provided by Aska Pharmaceuticals, Tokyo, Japan) for 7 days. The spheroids were fixed with 4% paraformaldehyde (PFA) and paraffin‐embedded blocks for histochemical analyses.

The ULM cells in 2D monolayer and 3D spheroid cultures were maintained in DMEM/F12 containing 2% charcoal‐stripped FBS (Sigma), 2 mM L‐glutamine, and 1% antibiotic‐antimycotic at 37°C in a humidified atmosphere of 5% CO_2_ and 95% air. The concentrations of E, P, and SPRM were 10^−8^ M, 10^−6^ M, and 10^−7^ M, respectively. The medium was exchanged every other day.

### 2.4 Evaluation of hormone sensitivity

In 2D monolayer cultures, the effect of female hormones was evaluated by a cell proliferation assay.^39^, ^40^ After 7 days of female hormone treatments, ULM cells were trypsinized to form a single‐cell suspension, and the number of viable cells was measured with a cell counter, Vi‐CELL XR (Beckman Coulter, Brea, CA). The cell number in each well of a 12‐plate was calculated. Incubations were performed in triplicate in a specimen of each ULM subtype. Four incubations of four specimens were performed, and data were shown as mean ± SD of 4 incubations.

In 3D spheroid cultures, the effect of female hormones was evaluated by the size and histology of the ULM spheroid after 7 days of female hormone treatment. The size of a spheroid was expressed as its largest cross‐sectional area. The cross‐sections were imaged with a Keyence BZ‐X710 microscope (Keyence, Osaka, Japan) and measured with ImageJ. Six spheroids were made from each ULM specimen. The mean of 6 spheroids was made as a value of a specimen. Data are shown as the mean ± SD of 5 specimens of MED12m(−)‐ULM and 9 specimens of MED12m(+)‐ULM.

### 2.5 Immunofluorescent staining of ULM cells in the 2D monolayer culture and spheroid sections

For 2D monolayer cultures, the immunocytochemistry of ULM cells cultured in a 12‐plate was done on day 1 after subculture according to the method as reported previously.^33^ Briefly, the cells were fixed with 4% PFA for 15 min, permeabilized with 0.5% triton‐X100, and blocked with a blocking solution (10% bovine fetal serum and 1% bovine serum albumin in PBST) for 60 min, incubated with an anti‐α‐SMA monoclonal antibody (Abcam, Tokyo, Japan; RRID: AB_262054), anti‐vimentin monoclonal antibody (Abcam, RRID: AB_10562134), anti‐ESR1 monoclonal antibody (Abcam, RRID: AB_1310196), or anti‐PGR monoclonal antibody (Abcam, RRID: AB_443421) as a primary antibody (diluted at 1:500) at 4°C overnight, incubated with the Alexa Fluor 488 conjugated anti‐mouse IgG (Abcam) or Alexa Fluor 594 conjugated anti‐rabbit IgG (Abcam) as a secondary antibody (diluted at 1:1000) for 45 min under dark condition, and counter‐stained with DAPI (Nacalai, Tokyo, Japan). The percentage of SMCs (α‐SMA positive cells) or PGR/ESR1 positive cells was calculated for six randomly chosen areas at ×200 magnification and expressed as mean ± SD.

For 3D spheroid cultures, immunohistochemistry was performed as reported previously.^8^, ^12^, ^41^, ^42^ Briefly, the sections (5 μm) of paraffin‐embedded spheroid samples were deparaffinized, fixed with 4% PFA for 5 min, permeabilized with 0.5% Triton‐X100 and blocked with blocking solution for 60 min, incubated with an anti‐α‐SMA monoclonal antibody or anti‐vimentin monoclonal antibody as a primary antibody at 4°C overnight, and incubated with the Alexa Fluor 488‐conjugated anti‐mouse IgG or Alexa Fluor 594‐conjugated anti‐rabbit IgG as a secondary antibody for 45 min, respectively. Nuclei were stained with DAPI.

### 2.6 Trichrome staining of ULM spheroid sections

Collagen fibers were visualized using a Trichrome Stain Kit (ScyTec Laboratories, West Logan, UT) according to the manufacturer's instructions.^8^, ^12^ The sections (5 μm) of paraffin‐embedded ULM spheroid samples were deparaffinized, fixed in Bouin's solution, stained with Weigert's Iron Hematoxylin for 10 min, immersed in Biebrich Scarlet‐Acid Fuchsin solution for 10 min, stained with Phosphomolybdic‐Phosphotungstic Acid for 15 min, stained with Aniline Blue for 15 min, and immersed in Acetic Acid (1%) for 1 min. The area of collagen fibers, which were stained blue, was quantified by ImageJ. The collagen fiber area (%) was calculated for 5 randomly chosen areas at ×200 magnification. The mean of the 5 randomly chosen areas was made as a value of a spheroid. Six spheroids were made from one ULM specimen. The mean of 6 spheroids was made as a value of a specimen. Data are shown as the mean ± SD of 3 specimens of MED12m(−)‐ULM and 3 specimens of MED12m(+)‐ULM.

### 2.7 TUNEL staining of ULM spheroid sections

Apoptotic cells were visualized using an In Situ Apoptosis Detection Kit (Takara) according to the manufacturer's instructions. The sections (5 μm) of paraffin‐embedded ULM spheroid samples were deparaffinized, permeabilized with 20 μg/mL proteinase K (Qiagen) for 15 min, intrinsic peroxidase‐blocked with 3% H_2_O_2_ for 5 min, incubated with terminal deoxynucleotide transferase at 37°C for 60 min, incubated with an anti‐FITC HRP‐conjugated antibody at 37°C for 30 min, incubated with diaminobenzidine (Wako) for 20 min, and counter‐stained with methyl green (Wako) for 20 min. Slides of paraffin‐embedded rat mammary glands included in the kit were used as a positive control of apoptotic cells. Apoptotic cells were checked in three specimens in each MED12m(−)‐ULM and MED12m(+)‐ULM.

### 2.8 Statistical analysis

The significance of differences was analyzed using the Kruskal–Walis, and Mann–Whitney tests. *p* values <0.05 were considered to indicate statistical significance. All statistical analyses were performed using the R software program (Ver 3.6.1).

## 3 RESULTS

### 3.1 Effects of female hormones on cell proliferation in 2D monolayer cultures of ULM cells

The number of viable cells after 7 days of female hormone treatments did not differ in any of the treatments in either of the ULM subtypes (Figure 1E).

### 3.2 Effects of female hormones on spheroids in 3D spheroid cultures of ULM cells

After 7 days of female hormone treatments, immunostaining of α‐SMA and vimentin was weak in the CTRL group, but the nuclei was clearly detected in the DAPI‐stained cells (Figure 2C). For both ULM subtypes, immunostaining for α‐SMA and vimentin were much stronger in the E + P and P groups than in the other groups (Figure 2C).

Spheroid sizes in the groups treated with female hormones (E + P, P, E, and E + P + SPRM) were significantly larger than those of the CTRL in both ULM subtypes (Figure 2D). Spheroid size in the E + P group was significantly larger than the sizes in the E and E + P + SPRM groups and similar to that in the P group (Figure 2D). Spheroid sizes in the E and E + P + SPRM groups were not significantly different (Figure 2D), indicating that the PGR inhibitory effects by SPRM are clearly shown in the ULM spheroid cultures. From these findings, the ULM spheroid culture system in this study is considered to be a culture system that can evaluate the responsiveness of ULM cells to female hormones independent of ULM subtypes.

### 3.3 Trichrome staining in ULM spheroids

**FIGURE 3:**
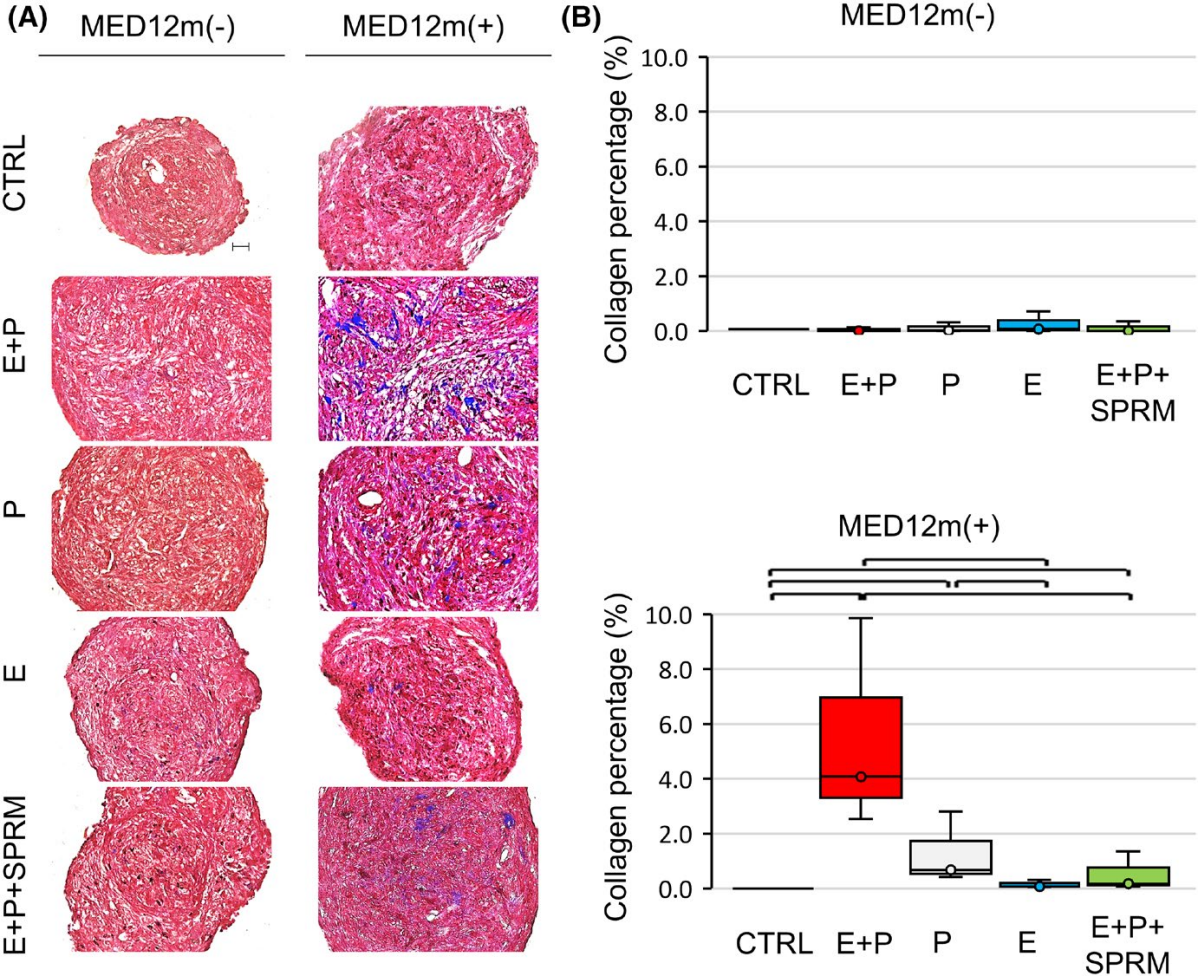
Detection of collagen fibers in ULM spheroids by trichrome staining. (A) Representatives of trichrome staining of the sections of each culture group in MED12m(−)‐ and MED12m(+)‐ULM spheroids after 7 days of hormone treatments. In trichrome staining, collagen fibers and cellular components are stained blue and red‐brown, respectively. Scale bar = 100 μm. (B) The area of collagen fibers was quantified by ImageJ. The collagen fiber area (%) was calculated on 5 randomly chosen areas at ×200 magnification. The mean of the 5 randomly chosen areas was a value of a spheroid. Six spheroids were made from a specimen of ULMs. The mean of 6 spheroids was a value of a specimen. Data Box plots show the mean ± SD of 3 specimens of MED12m(−)‐ULM and 3 specimens of MED12m(+)‐ULM. Brackets in the box plots indicate significant differences between the culture groups (*p*‐value < 0.05, Mann–Whitney test).

The collagen fibers in the ULM spheroids were histologically examined by trichrome staining. After 7 days of female hormone treatments, collagen fibers stained in blue were not detected in the spheroids of any culture groups in the MED12m(−)‐ULM (Figure 3A). On the other hand, in the MED12m(+)‐ULM, the collagen fibers were observed in the E + P, P, and E + P + SPRM groups, whereas they were not detected in the P‐free groups (E and CTRL) (Figure 3A). In addition, the collagen fiber content was significantly higher in the E + P group compared to the other groups in the MED12m(+)‐ULM (Figure 3B). These results suggest that the spheroid cultures produced more collagen fibers in the MED12m(+)‐ULM.

### 3.4 TUNEL staining in ULM spheroids

**FIGURE 4:**
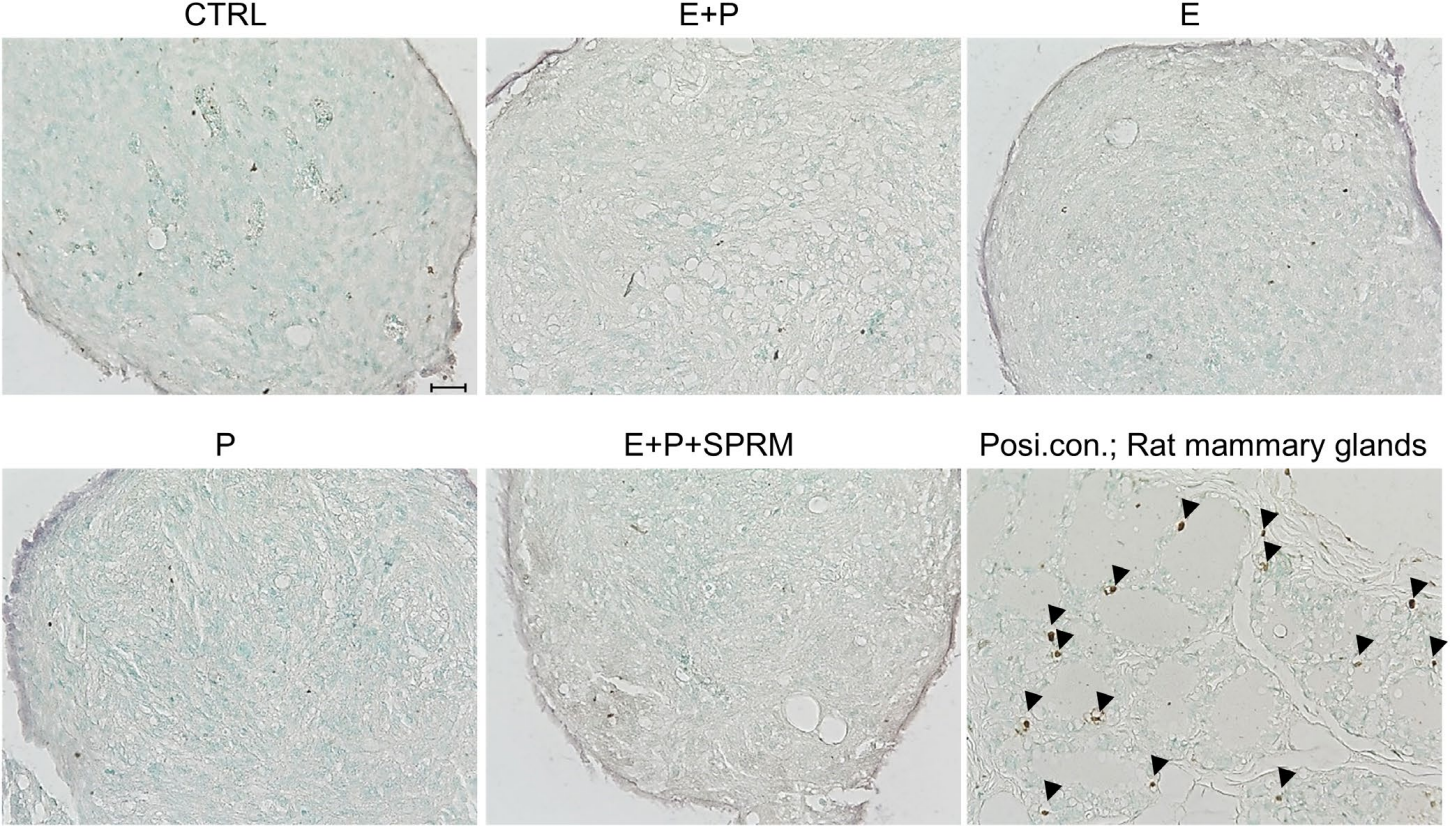
Detection of apoptotic cells in ULM spheroids by TUNEL staining. MED12m(−)‐ and MED12m(+)‐ULM spheroids after 7 days of hormone treatments were used. The images show the representatives of each culture group of the MED12m(−)‐ULM spheroid. Rat mammary tissue was used as a positive control to detect the apoptotic cells. In TUNEL staining, apoptotic cells were detected as the cells that bore dark red‐brown nuclei (arrowheads). Scale bar = 50 μm. Six spheroids were made from a specimen of ULMs. Apoptotic cells were checked in 5 randomly chosen areas at ×200 magnification in a spheroid. Three specimens were used in each MED12m(−)‐ULM and MED12m(+)‐ULM. Noted that, in both ULM subtypes, no TUNEL‐positive apoptotic cells were detected in any culture groups.

Apoptosis in ULM spheroids was histologically examined by TUNEL staining. In both ULM subtypes, no TUNEL‐positive apoptotic cells were detected in either culture group (Figure 4), suggesting that the difference in the spheroid size among the culture groups was not due to apoptosis of ULM cells.

## 4 DISCUSSION

In this study, we investigated the effect of female hormones on cell growth and survival in 2D monolayer and 3D spheroid cultures in each ULM subtype. In 2D monolayer cultures, since cell proliferation was not enhanced by female hormone treatments in either subtype, the effect of female hormones could not be evaluated. On the other hand, in 3D spheroid cultures, spheroid size was reduced in CTRL but was maintained with E + P or P. Thus, the 3D spheroid culture established in this study is more useful than a 2D culture to evaluate the effect of female hormones in vitro. A similar finding was also reported using an immortalized mouse fibroblast cell line, in which 3D spheroid cultures were more sensitive to estrogen than 2D cultures.^43^

Our results with 2D cultures were inconsistent with previous findings that ULM cells were proliferated by E, P, and E + P in monolayer cultures.^15^, ^16^, ^17^, ^18^ The discrepancy may be due to differences in culture conditions or cellular compositions. We used the collagen‐coated dish for the primary culture of ULM cells because collagen increases the proliferation activities of ULM cells by acting as cell scaffolds.^21^ In fact, the percentage of SMCs in the ULM cells was very high (83.7% and 82.7% in MED12m(−)‐ and MED12m(+)‐ULM, respectively) in this study, indicating that ULM cells in our study are predominantly SMCs. On the other hand, previous studies reported that the ratio of SMCs in ULM cells decreased during cell culture or through several passages, and consequently, most ULM cells were composed of FBs in a monolayer culture.^22^, ^23^ Furthermore, we confirmed the expression of PGR and ESR1 in the ULM cells of both ULM subtypes in this study. This suggests that the loss of PGR/ESR1 expression in ULM cells was not the reason why they did not respond to female hormones in the 2D culture. However, we cannot neglect the possibility that the cellular size may be altered by female hormones in our 2D culture.

The present study is the first to focus on the responsiveness of ULM cells to female hormones using ULM spheroids. Although there are several studies on cell senescence or survival using a 3D spheroid culture with primary‐cultured ULM cells,^26^, ^27^, ^28^ no studies have investigated the effect of female hormones on ULM cell growth in a 3D spheroid culture to date. Our 3D spheroid culture showed that the effect of female hormones can be evaluated by measuring the cross‐sectional area of the ULM spheroids in both ULM subtypes. In this study, to further examine the effect of P via PGR in spheroid cultures, we also investigated the effect of the SPRM (UPA). In E + P + SPRM, the spheroid sizes in both ULM subtypes were reduced in comparison to E + P and comparable to those of E, suggesting that the PGR inhibitory function of SPRM was clearly shown in our spheroid culture system. Although UPA is reported to induce apoptosis in ULM cells in 2D monolayer cultures, no TUNEL‐positive apoptotic cells were observed in any culture groups, including E + P + SPRM, in this culture system. As mentioned above, this discrepancy may be due to differences in cellular components and culture conditions between our study and the previous studies. The fact that we did not observe apoptotic cells in our culture system also suggests that the decrease in spheroid size was due to a shrinkage of individual cells, but not to a reduction of cell number. Our results appear to be consistent with previous findings using a xenograft model in which the progesterone receptor antagonist RU486 (mifepristone) or female hormone withdrawal caused a decrease in the size of individual cells, rather than a reduction in cell number via cell death.^13^, ^44^ A limitation of the present study was that we were unable to measure the sizes of individual cells because of their high cellular density.

In the 3D spheroid cultures, it is interesting that collagen fiber production was greater in the MED12m(+)‐ULM. Collagen fibers were only observed in the group with P‐addition in the MED12m(+)‐ULM spheroids. The difference in the capacity of collagen production between ULM subtypes is also evident from the histological composition of the ULM tissue in vivo, in which collagen fibers occupy almost half of the tumors in MED12m(+)‐ULM while collagen fiber contents are much less abundant in MED12m(−)‐ULM.^11^, ^12^ In addition, by comparing transcriptomes, we recently showed that the signaling pathways related to ECM production are activated in MED12m(+)‐ULM and inactivated in MED12m(−)‐ULM compared to normal myometrium.^12^ Therefore, our 3D spheroid culture system is likely to reproduce the difference in collagen fiber production between the two ULM subtypes. In addition, our results revealed that the difference in collagen production between the two ULM subtypes was P‐dependent in this culture system. We speculate that this difference may be due to the interaction between progesterone and MED12 mutant protein in MED12m(+)‐SMCs, as previously reported in the altered regulation of *RANKL* gene expression by MED12 mutant protein and PG.^45^, ^46^ It is not surprising that collagen fiber was produced in the E + P group because estrogen is necessary to induce progesterone receptors so that progesterone can act.^13^

In this study, we established a 3D spheroid culture system that can evaluate the responsiveness of ULM cells to female hormones. This culture system may be a useful tool for further elucidating the pathogenesis of ULMs and screening effective drugs for ULMs.

## FUNDING INFORMATION

This work was supported in part by ASKA Pharmaceutical Co., Ltd., and JSPS KAKENHI Grants (JP24K12533, JP24K12579, JP24K12626, JP24K19699, JP24K23514, JP23K27734, JP23K08889, JP23K08824, JP23K07312, JP23K15838, JP22K19603, JP22K09620) for Scientific Research from the Ministry of Education, Science, and Culture, Japan.

## CONFLICT OF INTEREST STATEMENT

Sakai T, Sato S, Tamehisa T, and Sugino N received research funding from ASKA Pharmaceutical Co., Ltd. Sugino N is an editorial board member of Reproductive Medicine and Biology and a co‐author of this article. To minimize bias, he was excluded from all editorial decision‐making related to the acceptance of this article for publication. The other authors declare that there are no conflicts of interest.

## ETHICS STATEMENT

All experiments involving human tissues were conducted according to the protocol approved by the Institutional Review Board of Yamaguchi University Graduate School of Medicine (No. H27‐035).

## HUMAN RIGHTS STATEMENTS AND INFORMED CONSENT

All experiments involving human tissues were conducted according to the Declaration of Helsinki. Before sample collection, informed consent was obtained from all patients.
